# Visualization of new vessel formation during venous thrombus organization using genetic fate mapping

**DOI:** 10.1016/j.rpth.2026.106842

**Published:** 2026-07-14

**Authors:** Magdalena L. Bochenek, Julie D. Noorali, Aden Aderajew, Kateryna Moiko, Stefanie Zimmer, Konstantinos Zifkos, Mathias Wagner, Philipp Lurz, Lukas Hobohm, Katrin Schäfer

**Affiliations:** 1 Department of Cardiology, University Medical Center, Johannes Gutenberg University, Mainz, Germany; 2 Deutsches Zentrum für Herz-Kreislauf-Forschung (DZHK) e.V., partner site RhineMain, Mainz, Germany; 3 Center for Thrombosis and Hemostasis (CTH), University Medical Center, Johannes Gutenberg University, Mainz, Germany; 4 Institute of Pathology, University Medical Center, Johannes Gutenberg University, Mainz, Germany; 5 Institute of Pathology, Saarland University Medical Center and Saarland University Faculty of Medicine, Homburg, Germany

**Keywords:** endothelial cells, genetic fate mapping, remodelling, revascularization, venous thrombosis

## Abstract

**Background:**

Endothelial cell (EC) dysfunction contributes to venous thrombosis, and disrupting endothelial angiogenic signaling delays venous thrombus resolution. However, the time course of EC appearance during venous thrombus organization and the identity of cells lining the intrathrombotic vessels are largely unknown.

**Objectives:**

We used a genetic fate mapping approach to visualize tamoxifen-inducible endothelial receptor tyrosine kinase (TIE2) reporter ECs during venous thrombus resolution and human pulmonary thrombi of different organizational stages to validate our findings.

**Methods:**

We induced stenosis and deep vein thrombosis by ligating the inferior vena cava in mice expressing membrane targeted green fluorescent protein in postembryonic TIE2 lineage cells. TIE2 reporter ECs started to appear on day 7 and lined the newly formed vessel-like structures within thrombi on day 21.

**Results:**

Immunolabeling identified endomucin as most specific marker of angiogenic ECs in venous thrombi. Intercellular adhesion molecule 2, a marker of EC activation, was expressed predominantly on ECs lining the adjacent aorta, not the inferior vena cava. Cells expressing the hypoxia surrogate marker carboanhydrase IX, stromal cell-derived factor 1 alpha, or CD34 peaked on day 7, whereas endothelial protein C receptor–expressing cells appeared later. Platelet-derived growth factor beta lineage–derived pericytes and fibrosis were detected from day 14 onwards. Histologic analysis of thrombus material from patients with acute pulmonary embolism or chronic thromboembolic pulmonary hypertension confirmed the presence of endomucin-, CD34-, and endothelial protein C receptor–positive cells in organized pulmonary thrombus material, whereas vessel-like structures were only seen in chronic thromboembolic pulmonary hypertension thrombi.

**Conclusion:**

TIE2 reporter ECs appear in venous thrombi early and prior to measurable reductions in thrombus size, whereas pericytes appear later and possibly contribute to vessel stabilization.

## Essentials

• The formation of new blood vessels that canalize venous thrombi helps to restore blood flow.
• Thrombus recanalization was studied in mice with genetically labeled vascular or blood cells.
• Endomucin-positive, TIE2 reporter endothelial cells appear first, and pericytes appear later.
• Our time course analysis may aid the refinement of revascularization strategies in humans.

## Graphical abstract

**Figure:**
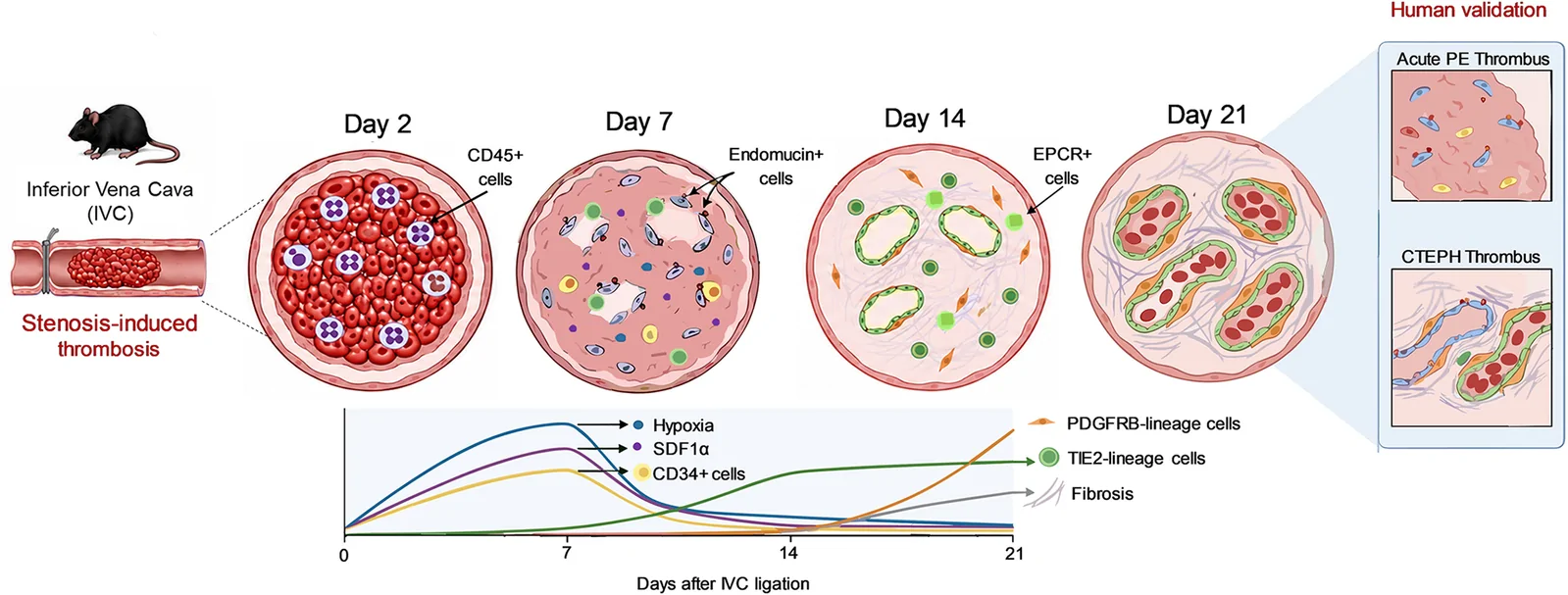

## 1 Introduction

The importance of healthy or functional endothelial cells (ECs) in the prevention of platelet aggregation and leukocyte activation and in the maintenance of a nonthrombogenic vascular environment is well known [1], and endothelial dysfunction is an established risk factor for thrombus formation, in both the arterial and the venous system [[2], [3], [4]]. Moreover, and in addition to factors of the fibrinolytic system produced by immune and vascular cells, ECs also contribute to thrombus resolution and the restoration of vessel patency by driving thrombus revascularization [5,6]. The concept of neovascularization in venous thrombus resolution has emerged already >25 years ago [7], and work from us and others has shown that experimental disruption of endothelial angiogenic signaling delays venous thrombus resolution [5,8]. Conversely, antiangiogenic therapy was found to inhibit or delay thrombus resolution [9,10]. Regarding specific angiogenic growth factors during venous thrombus resolution, experimental work revealed central roles for not only vascular endothelial growth factor (VEGF) [11,12] or interleukin 8 [13] but also factors that impair angiogenesis, in particular transforming growth factor β) [14] or angiopoietin 2 [15]. In addition, loss-of-function studies in mice showed that molecules expressed by ECs, such as platelet endothelial cell adhesion molecule 1 (also known as CD31), are more than just endothelial markers and have active roles during venous thrombus resolution [16].

However, and despite all progress made so far, important questions remain unanswered, including the origin of the ECs within the thrombi and whether they are derived from the vein wall or the circulating blood. It is also unclear at which time during thrombus organization that ECs migrate into the clot and whether they follow pericytes or invade the thrombus first. The relevance of these questions is underlined by disease phenotypes driven by the failure of ECs to revascularize thrombi, a prominent and clinically relevant example being chronic thromboembolic pulmonary hypertension (CTEPH) [17,18].

In this study, we used a lineage-tracing approach to genetically fate-map tamoxifen-inducible endothelial receptor tyrosine kinase (TIE2) lineage–derived ECs and their progenitors, as well as platelet-derived growth factor receptor beta (PDGFRB) lineage pericytes in mice, and to follow their presence at different time points after stenosis-induced thrombosis in deep veins. The clinical relevance and significance for venous thrombus organization in humans is demonstrated by the analysis of key markers in pulmonary embolectomy and endarterectomy tissue samples from patients with acute pulmonary embolism (PE) or CTEPH, as well as in postmortem lung tissue samples from patients who did not survive venous thrombosis.

## 2 Methods

### 2.1 Experimental animals

To genetically label cells of the endothelial, mesenchymal, and erythrocyte lineage, we generated 3 reporter mouse lines using the Cre-loxP system. To fate-track early ECs, we used transgenic mice expressing Cre recombinase under control of the TIE2 promoter (TIE2.CreER^T2^; Tg[Tek-cre/ERT2]1Arnd; MGI-ID: 2450312; kind gift of Bernd Arnold, DKFZ Heidelberg) [19]. To fate-track mesenchymal cells, mice expressing Cre recombinase under control of the tamoxifen-inducible PDGFRβ promoter were used (PDGFRB.CreER^T2^; B6.Cg-Tg[Pdgfrb-cre/ERT2]6096Rha/J; MGI-ID: 236849 [20]; Jackson Laboratories; strain 029684; RRID:IMSR_JAX:029684). Cre recombinase activity was induced by feeding 5-week-old TIE2.CreER^T2^ and PDGFRB.CreER^T2^ mice standard rodent diet containing tamoxifen citrate (400 ppm; TD55125; Harlan Teklad) for 5 weeks, followed by a 2-week-long tamoxifen washout phase. To visualize erythrocytes, mice constitutively expressing Cre recombinase under the erythropoietin receptor (EpoR) promoter, expressed by early erythroid progenitor cells, were used (EpoR-Cre; B6.C-Eportm1.1[EGFP/icre]Uk/MdfJ; MGI-ID: 92235; kind gift of Ursula Klingmüller, DKFZ, Heidelberg, Germany) [21]. Male Cre recombinase transgenic mice of the abovementioned strains were crossbred with female ROSA26mTmG transgenic mice ([B6.129(Cg)-Gt(ROSA)26Sortm4(ACTB-tdTomato,-EGFP]Luo/J; MGI-ID: 124702 [22]; Jackson Laboratories; strain 007676; RRID:IMSR_JAX:007676). This resulted in irreversible genetic labeling of Cre recombinase–expressing cells with membrane enhanced green fluorescent protein (mEGFP) and allowed us to perform lineage tracing experiments *in vivo*. Genotyping was performed using genomic DNA isolated from ear biopsies using the specific primers shown in Supplementary Table 1. Age-matched Cre transgenic (tg) male littermate mice were used throughout the study. All experiments in mice are reported according to the ARRIVE (Animal Research: Reporting of *In Vivo* Experiments) guidelines [23] and were carried out in accordance with the U.K. Animals (Scientific Procedures) Act, 1986, and associated guidelines.

### 2.2 Mouse model of stenosis-induced deep vein thrombosis

Inferior vena cava (IVC) ligation was performed, as described [14,24]. Shortly, male mice (12 weeks old) were anesthetized via intraperitoneal injection of a mixture of fentanyl (Fentadon; Dechra; 0.05 mg/kg body weight [BW]), medetomidin (Dorbene vet; Zoetis; 5.0 mg/kg/BW), and midazolam (5.0 mg/kg/BW). Mice were placed on a prewarmed (37 °C) heating pad (NC9764401; Thermo Scientific). The IVC was carefully dissected immediately caudal of the left renal vein and a thread (6-0 silk suture; 41542N; Resorba) was used to ligate the IVC. To allow minimal blood flow, a placeholder (5-0 Prolene suture; 70990; Ethicon) was placed on the anterior surface of the IVC and removed immediately after the ligation to allow minimal blood flow. Back and side branches were not ligated [25]. At the end of the procedure, the surgical incision was closed with a Prolene suture (6-0; 8695G; Ethicon), and the sedation was reversed by intraperitoneal injection of a mixture of atipemazol (Antisedan; CP Pharma; 0.05 mg/kg/BW) and flumazenil (Flumazenil Inresa; 0.01 mg/kg/BW). Buprenorphine hydrochloride (Bupresol vet Multi; CP Pharma; 0.1 mg/kg/BW) was subcutaneously injected immediately following the procedure and on day 1 and day 2. Only male mice were used because anatomical differences between male and female mice may affect venous thrombosis in this model and lead to ischemic complications in females [26]. All animal procedures were included in the animal license permit G19-1-092.

### 2.3 High-resolution vascular ultrasound

To determine the extent of venous thrombus formation and the restoration of blood flow following surgery, mice were anesthetized via 2.5% isoflurane gas inhalation (Piramal Critical Care; 66794-017-25) on days 2, 7, 14, and 21, and noninvasive hemodynamic measurements were performed using high-frequency ultrasound (Vevo 3100; Visual Sonic; FujiFilm) and a 55-MHz mouse scanhead (Visual Sonic; FujiFilm). The thrombus was outlined on an optimal freeze-frame image and its shortest and longest diameter measured and the cross-sectional area calculated using Vevo 770 software (Visual Sonic).

### 2.4 Blood and tissue harvest

At different time points after the IVC ligation, mice were anesthetized via isoflurane (2.0 vol%, Piramal Critical Care; 66794-017-25), and whole blood was drawn by cardiac puncture into 0.1 volume of 3.8% sodium citrate (Sigma-Aldrich; S4641). Isoflurane anaesthesia was then increased to 4.0 vol%, and deeply anesthetized mice were killed by cervical dislocation. Whole blood cell counts were determined using automated hematology analyzer (ABAXIS Vetscan VS2). The thrombosed IVC segment was excised, fixed with 4% paraformaldehyde (1004968350; Sigma-Aldrich) for 2 hours. This was followed by overnight incubations in first 15% and then 30% sucrose (57-50-1; Carl Roth) and embedding in Tissue-Tek O.C.T compound (4583; Sakura). Tissues were cut into 5-μm-thick serial longitudinal sections. For each thrombus, 3 sections, equally spaced through the thrombosed vein segment, were stained using modified Carstairs’ stain [27] to distinguish red blood cells (RBCs; yellow), fibrin (orange-red), and fibrosis (blue). Images were acquired using an Olympus BX51 light microscope, and the section containing the maximal thrombus area was selected for morphometric analysis, which was performed using Image ProPlus image analysis software (version 7.0). Staining intensity was quantified using the count-size function, and results were expressed as percentage of positively stained area relative to the total area of the microscopic field to account for differences in thrombus sizes. The person who acquired the images was separate from the person who performed the image analysis.

### 2.5 Fluorescence microscopy and image analysis

Immunofluorescence staining were performed on 8-μm-thick longitudinally cut, serial sections of cryopreserved tissues immediately adjacent to the section containing the maximal venous thrombus, identified as described earlier. Immediately before the staining, sections were thawed for 15 minutes at room temperature, permeabilized using 0.025% Triton X-100 (3051.3; Carl Roth) for 10 minutes at 37 °C and overnight incubated with primary antibodies. Antibodies against CD31 (SantaCruz Biotechnology; sc-18916 Mec13.3, RRID: AB_627028; dilution, 1:100) and VE-cadherin (ab33168, abcam; RRID: AB_870662) were used as general EC markers. Antibodies against CD34 (clone MEC14.7; abcam; ab8158; RRID: AB_306316; dilution, 1:100) and endothelial protein C receptor (EPCR; ThermoFisher Scientific; PA5-32217; RRID: AB_2549690; dilution, 1:100) were used to label hematopoietic stem and endothelial progenitor cells (EPCs). Antibodies against endomucin (eBiosciences; #14-5851-81, clone V.7C7; RRID: AB_891529; dilution, 1:50) to label venous ECs and antibodies against ICAM2 (Biozol; 1925-01; RRID: AB_2795538; dilution, 1:100) to label activated ECs. ECs were also visualized using isolectin B4 (Vector; FL1201; RRID: AB_2314663; 1:25). Hypoxia was visualized using carboanhydrase (CA) IX as surrogate marker (clone H-120; SantaCruz Biotechnology; sc-25599; RRID: AB_2066539; dilution, 1:25), inflammation by staining CD45-positive leukocytes (clone 30F11; SantaCruz Biotechnology; sc-53665; RRID: AB_629093; dilution, 1:100), chemoattractant expression by visualizing stromal-derived factor 1 alpha (SDF1α)-positive cells (#3740; Cell Signaling Technology; RRID: AB_2292511; dilution, 1:100). Then, goat antirabbit Alexa-647 (A32733; Invitrogen; dilution, 1:500) and goat antirat Alexa-647 (ab150159; abcam; dilution, 1:500) conjugated secondary antibodies were added for 1 hour at room temperature. Cell nuclei were visualized using 4′,6-diamidine-2-phenylindole (DAPI; Carl Roth; 6335.1), diluted 1:5000 in phosphate-buffered saline. Background was determined on slides incubated with secondary antibodies alone. Sections were covered with fluorescence mounting medium with background reducing properties (S3023; Dako) and photographed on a BZ-X800E microscope (Keyence Corporation). Green fluorescent reporter cells were detected without secondary antibodies.

For quantification, images were taken from the outer thrombus layer (including the vein wall and lumen) and the thrombus core, an example is shown in Supplementary Figure 1A. Adjacent, neighboring images were taken to cover the entire thrombus. Depending on the thrombus size, up to 15 images per area of interest were taken. For reporter-positive cells under control of Tie2.CreER^T2^ and PDGFRB.CreER^T2^, the number of reporter-positive cells was manually counted and expressed per total DAPI-positive cell number in the same microscopic field. Of note, only completely enclosed channels with a visible lumen and lined by cells expressing endothelial markers were counted as vessel-like structures. For reporter-positive cells under control of ErGFP.Cre, where erythrocytes do not contain nuclei, the staining intensity was quantified using ImageJ software (National Institutes of Health). The number of CA IX–, CD34-, CD45-, EPCR-, and SDF1α-immunopositive cells was manually counted and expressed as percentage of positive cells per total number of cells.

### 2.6 Flow cytometry

To determine the number of reporter-positive cells in TIE2.CreER^T2^ or PDGFRB.CreER^T2^ mice, lungs and aorta were isolated and digested using collagenase type A (for lungs) and collagenase type II (for aorta) for 30 minutes at 37 °C with agitation. The digested tissue was filtered through a 70-μm strainer (Greiner) and fixed using 0.1% paraformaldehyde. The number of mEGFP-positive cells in ErGFP reporter mice was measured in whole blood. The number of mEGFP-positive cells was determined using FACsCanto II (BD Biosciences) and is shown as percentage of the total number of cells.

### 2.7 Intravital microscopy

To visualize the interactions between ECs lining the IVC and circulating blood cells (erythrocytes, platelets, or leukocytes), intravital microscopy was used, as described [24]. In brief, anesthetized mice were placed on a 37 °C heating pad, and a catheter (NC9764401; Thermo Scientific) was implanted in their right jugular vein. To detect erythrocytes, Cre wild-type (WT) membrane tomato (mT) ROSA26mTmG transgenic mice were used, in which all cell membranes are labeled by red fluorescence. Anticoagulated whole blood was drawn, and erythrocytes were isolated using density gradient centrifugation, as described [28]. To visualize platelets, rhodamine B (Sigma-Aldrich; R6626; 0.1 mg/kg/BW in 100 μL) was used; to visualize leukocytes, rhodamine-6G (Sigma-Aldrich; 83697; 0.1 mg/kg/BW in 100 μL) was used and slowly infused through the catheter shortly before the IVC ligation. Data were acquired in 30-minute intervals for up to 2 hours. To obtain and analyze the images, an upright microscope (Nikon) utilizing a spinning disk unit (CSU-W1; Yokogawa) and equipped with a sCMOS 4.2-megapixel camera (4.2 plus; Andor), water immersion objective (CFI-75 WD 16X; numerical aperture 1; Nikon), and LED light engine (SOLA SE II; Lumencolor) and the real-time imaging system NIS Elements software (Nikon) were used. Cell recruitment was quantified in 3 viewing fields per mouse, and the results were averaged. Adherent cells were defined as cells that did not detach for 100 frames. Adherent and rolling cells are reported as cells per microscopic view.

### 2.8 Human pulmonary thrombus material

Thrombotic material occluding the pulmonary artery was obtained from patients with acute PE (*n* = 4) undergoing percutaneous pulmonary embolectomy at the Department of Cardiology, University Medical Center Mainz, Germany (ethics approval AZ 2025-18273-bio), or from patients diagnosed with CTEPH (*n* = 4) who underwent pulmonary endarterectomy from the Institute of Pathology, Saarland University Medical Center and Saarland University Faculty of Medicine, Homburg, Germany (ethics approval Kenn-Nr. 88/22). All patients, who underwent pulmonary embolectomy or endarterectomy, gave their written informed consent prior to the intervention. Lung tissue from patients who died after PE (*n* = 4) was obtained from the Tissue BioBank at the Department of Pathology, University Medical Center, Mainz, Germany, and from the Institute of Pathology, Saarland University Medical Center and Saarland University Faculty of Medicine, Homburg, Germany (ethics approval Kenn-Nr. 88/22).

Human PE, pulmonary endarterectomy (PEA), and lung specimens were paraffin embedded and cut into 5-μm-thick serial cross-sections and deparaffinized and stained using hematoxylin and eosin to detect nucleated cells or using modified Carstairs’ stain [27] to distinguish RBCs (yellow-orange), fibrin (orange-red), and fibrosis (blue). Sirius red staining was used to detect interstitial collagens. Endomucin-positive ECs were detected using immunohistochemistry (Antibody Online; ABIN1385912; RRID: AB_11186816; dilution, 1:200); EPCs using CD34 (abcam; ab8158; clone MEC14.7, RRID: AB_306316; dilution, 1:20) and EPCR (Novus Biologicals; AF2245; RRID: AB_355196; dilution 1:40); and hypoxia using carboanhydrase IX (CA IX) as a surrogate marker (SantaCruz Biotechnology; sc-25599; clone H-120; RRID: AB_2066539; dilution, 1:25). Images were collected on an Olympus BX51 light microscope, and morphometric analyses were performed using Image ProPlus software (version 7.0). The intensity of the staining signal was measured using the count-size function and expressed as percentage positive per total area. For each region of interest, 4 embolectomy, 4 PEA specimens, and 4 lung specimens and 8 to 15 areas per tissue (depending on its size) were quantified and the results averaged per specimen. The results are shown as immunopositive area per total area for immunohistochemistry or the number of nucleated cells per total area for hematoxylin and eosin and endomucin.

### 2.9 Statistical analysis

Results are shown as mean ± SEM if normally distributed or as median with IQR if not. Normality and Lognormality was determined using the Shapiro–Wilk test. If 2 groups were compared, Student’s *t*-test was used for normally distributed values. If >2 groups were compared, 1-way analysis of variance (anova) followed by Sidak multiple comparison test was used for normally distributed values and Kruskal–Wallis followed by Dunn multiple comparison test for nonnormally distributed values. More than 2 groups and an additional variable were compared using 2-way anova. All data were analyzed with GraphPad Prism, version 10.4.1 (GraphPad Software).

## 3 Results

### 3.1 TIE2 endothelial reporter cells appear on day 7 in the outer thrombus layers and line intrathrombus vessels on day 21

**Figure 1:**
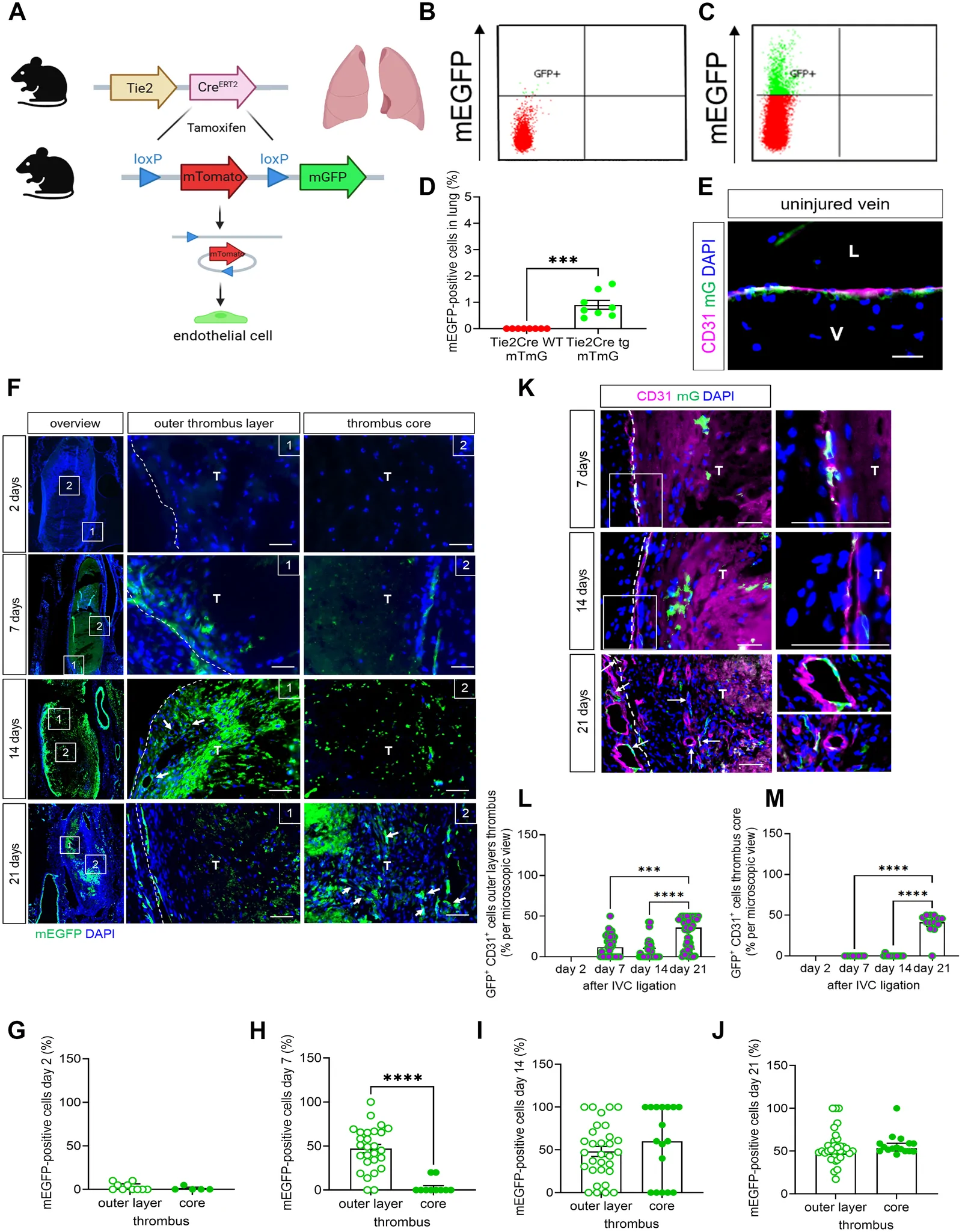
Time course of tamoxifen-inducible endothelial receptor tyrosine kinase (TIE2)-positive endothelial cell presence in experimental venous thrombi during thrombus organization. Schematic drawing showing the experimental work flow using design of tamoxifen-inducible endothelial (TIE2.ER^T2^) reporter mouse (A). Representative flow cytometry dot blots showing TIE2 reporter cells isolated from the lung of TIE2.ER^T2^ wild-type (WT) mTmG (B) and TIE2.ER^T2^ TG mTmG (C) mice. (D) The results of the quantitative analysis. Representative fluorescence image of cryopreserved uninjured IVC from a TIE2.ER^T2^ TG mTmG mouse immunostained using CD31 antibodies (purple signal) (E). Representative images (F) and quantitative analysis (G–J) of venous thrombus isolated at different time points (as indicated) for the relative number of mEGFP-positive cells. Representative images (K) and quantitative analysis of mEGFP-CD31-dual positive cells in the outer thrombus layer (L) and the thrombus core (M) at different time points. Right panel shows zoom-in images of the vessel wall. Scale bars in (E), (F), and (K): 100 μm. ∗∗∗*P* < .001; ∗∗∗∗*P* < .0001; Mann–Whitney U-test in (D) and (G–J) and Kruskal–Wallis, followed by Dunn multiple comparison test, in (L) and (M). Nonsignificant differences are not shown.

To visualize ECs during venous thrombus formation and resolution, we used a lineage tracing approach (Figure 1A). To genetically fate-map ECs while avoiding TIE2-mediated gene deletion in hematopoietic cells, we used mice expressing mTmG under the tamoxifen-inducible TIE2 promoter [19]. Tamoxifen induction was started in adult mice, at which time TIE2 promoter activity on hematopoietic stem cells is naturally weak [29,30], minimizing the possibility of reporter gene expression in hematopoietic stem cells and their progeny, as verified earlier by us [31] and others [19]. Flow cytometry analysis of whole lungs confirmed reporter-positive cells in TIE2.CreER^T2^ transgenic mTmG mice and the absence of signals in TIE2.CreER^T2^ WT mTmG mice (Figure 1B–D). Fluorescence microscopy documented reporter gene–expressing cells lining the inner lumen of the uninjured IVC, and costaining for CD31 confirmed their endothelial identity (Figure 1E). Following the IVC ligation, TIE2 reporter ECs were not present on day 2, started to appear on day 7, and were clearly visible in larger numbers on days 14 and 21 (Figure 1F, G). TIE2 reporter ECs were predominantly detected within the outer layers of the thrombus close to the vein vessel wall on day 7 (Figure 1H and Supplementary Table 2), whereas their numbers in the thrombus core increased on days 14 and 21 (Figure 1I, J). TIE2 reporter ECs expressing CD31 started to appear at day 7, primarily at the outer layers of the thrombus, and their numbers increased over time (Figure 1K–M). However, intrathrombotic vessel-like structures lined by CD31-positive TIE2 reporter ECs were not seen before day 21.

Ultrasound time course analysis of venous thrombus organization over 3 weeks (Supplementary Figure 2A) revealed that the thrombus area did not change until day 14 (Supplementary Figure 2B, C) and that the reduction in thrombus area seen on day 21 was due to significant changes in thrombus length (Supplementary Figure 2D), whereas the thrombus diameter did not change (Supplementary Figure 2E). Modified Carstairs’ staining to visualize changes in venous thrombus composition showed that the fibrin-positive area decreased over time (a significant reduction compared with day 2 was seen on days 14 and 21) (Supplementary Figure 2F). At the same time, an increase in fibrotic tissue was observed, reaching statistical significance on day 21 (Supplementary Figure 2G). Thus, TIE2 reporter ECs can be observed within the experimental venous thrombi before changes in thrombus size and organization from fibrin to fibrosis are measurable by vascular ultrasound or histology.

### 3.2 The revascularization of venous thrombi occurs via endomucin-expressing venous endothelial cells

**Figure 2:**
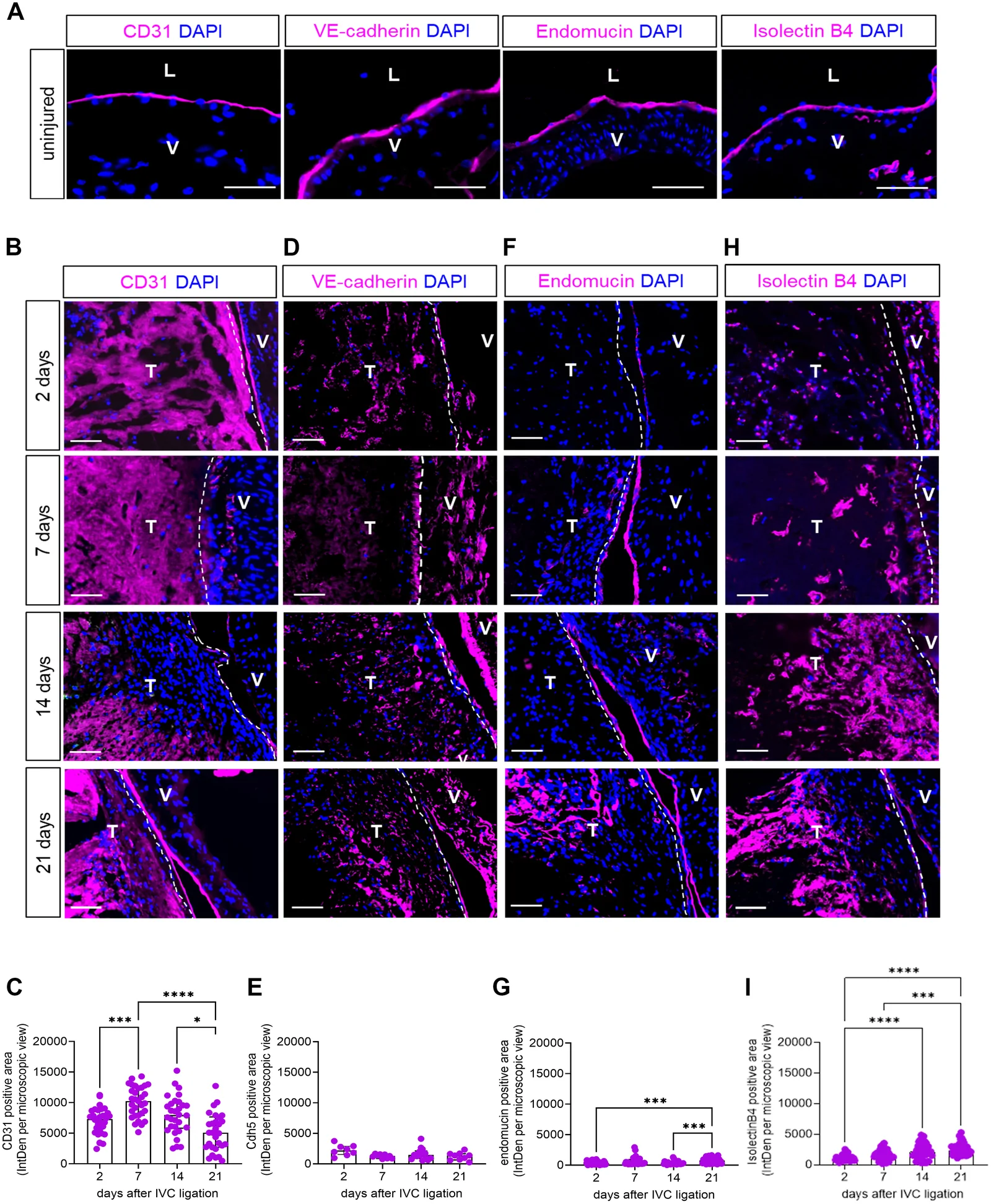
Characterization of endothelial cells residing in venous thrombus identifies endomucin as an early and specific marker for ECs revascularizing the venous thrombi. Representative images of the uninjured (A) and thrombosed inferior vena cava (IVC; B, D, F, H) stained for CD31, VE-cadherin, endomucin, and isolectin B4. Cell nuclei are labeled using 4′,6-diamidine-2-phenylindole (DAPI). Representative images and the results of the quantitative analysis of venous thrombi isolated at different time points (as indicated) and stained for CD31 (B and C), VE-cadherin (D and E), endomucin (F and G), and isolectin B4 (H and I) are shown. V indicates the vein; L, the lumen between vein wall and thrombus, and T, the thrombus. A scattered white line was added to outline the thrombus borders. Scale bars in (A) and (B): 100 μm. ∗*P* < .05, ∗∗∗*P* < .001, ∗∗∗∗*P* < .0001; 1-way anova, followed by Sidak multiple comparison test, in (E) and Kruskal–Wallis, followed by Dunn multiple comparison test, in (C), (G), and (I). Nonsignificant differences are not shown.

We already observed that TIE2 reporter ECs express the endothelial cell marker CD31 (Figure 1K–M). To better characterize ECs lining the vessel-like structures within the venous thrombi and to establish the optimal marker to detect different types of ECs and changes in their expression patterns following the IVC ligation, we tested antibodies against endothelial antigens. ECs lining the inner layer of the uninjured IVC, the site of venous thrombus formation in our model, were immunopositive for markers commonly used to detect ECs, that is CD31 (Figure 2A), expressed on early and mature ECs, as well as on platelets and some leukocyte subtypes [32]. They also were not only positive for the tight junction protein VE-cadherin, often used as marker for mature ECs, but also expressed on non-ECs such as perineurial cells [33]. In addition, they stained positive for endomucin, a glycoprotein with antiadhesive properties highly expressed on postcapillary venules [34]. A specific visualization of ECs was also possible using Griffonia simplicifolia isolectin (I)B4, which binds to specific carbohydrates on rat and murine ECs [35] via nonimmunologic mechanisms. However, and while cells lining the inner surface of the uninjured IVC showed strong, continuous, and specific staining signals for all the abovementioned markers, their expression patterns changed following the IVC ligation.

CD31-immunopositive cells were still seen lining the inner surface of the IVC on day 2, were reduced on days 7 and 14, and reappeared on day 21 (Figure 2B). On the contrary, large amounts of CD31-immunopositive material, often without the vicinity of DAPI-positive cells was present within the thrombus at all time points and peaking at day 7 (Figure 2B, C), in line with the known expression of CD31 by anucleated platelets [32]. Platelets adhering to denuded inner IVC layer probably also explain the presence of strong CD31 immunosignals in the absence of TIE2 reporter ECs at this site. VE-cadherin–immunopositive cells also were reduced in numbers in the inner layer of IVC on day 2 and reappeared on day 7 (Figure 2D). However, VE-cadherin expression did not change within the thrombus over time (Figure 2E). Endomucin-immunopositive cells also were reduced on day 2 and reappeared as early as day 7, and a continuous lining of endomucin-positive cells was observed on days 14 and 21, very similar to that seen in uninjured veins (Figure 2F, G). Endomucin-immunopositive cells also were seen covering the outside of the thrombus and lining the vessel-like structures inside the thrombi on day 21 after the IVC ligation. Although the number of endomucin-immunopositive signals was lower than for CD31, the signal was more specific and restricted to nucleated cells lining vessels. IB4-positive cells were not visible from day 2 until day 14 at which time point they started to reappear, albeit at lower levels than the uninjured IVC (Figure 2H). The IB4-positive material within the thrombus significantly increased over time after the IVC ligation (Figure 2I), similar to endomucin. Supplementary Tables 3 and 4 show the results of the semiquantitative grading of the staining signals.

These analyses revealed the venous endothelial marker endomucin as an early and specific marker for ECs reendothelializing the ligated IVC and revascularizing the venous thrombi in our model. Interestingly, immunofluorescence microscopy analysis of the endothelial activation marker ICAM2, involved in leukocyte recruitment and the regulation of angiogenesis [36], revealed no changes on venous ECs lining the inner layer of the IVC, but an upregulated expression on ECs lining the adjacent aorta, most strongly on day 2 after the ligation (Supplementary Figure 3A). On day 21, ICAM2-expressing cells were also found lining small vessels in the adventitia and perivascular tissue in-between the IVC and the aorta. These findings suggest that the IVC ligation also activates ECs on neighboring nonvenous vessels.

### 3.3 CD34- and EPCR-positive cells participate in venous thrombus revascularization

**Figure 3:**
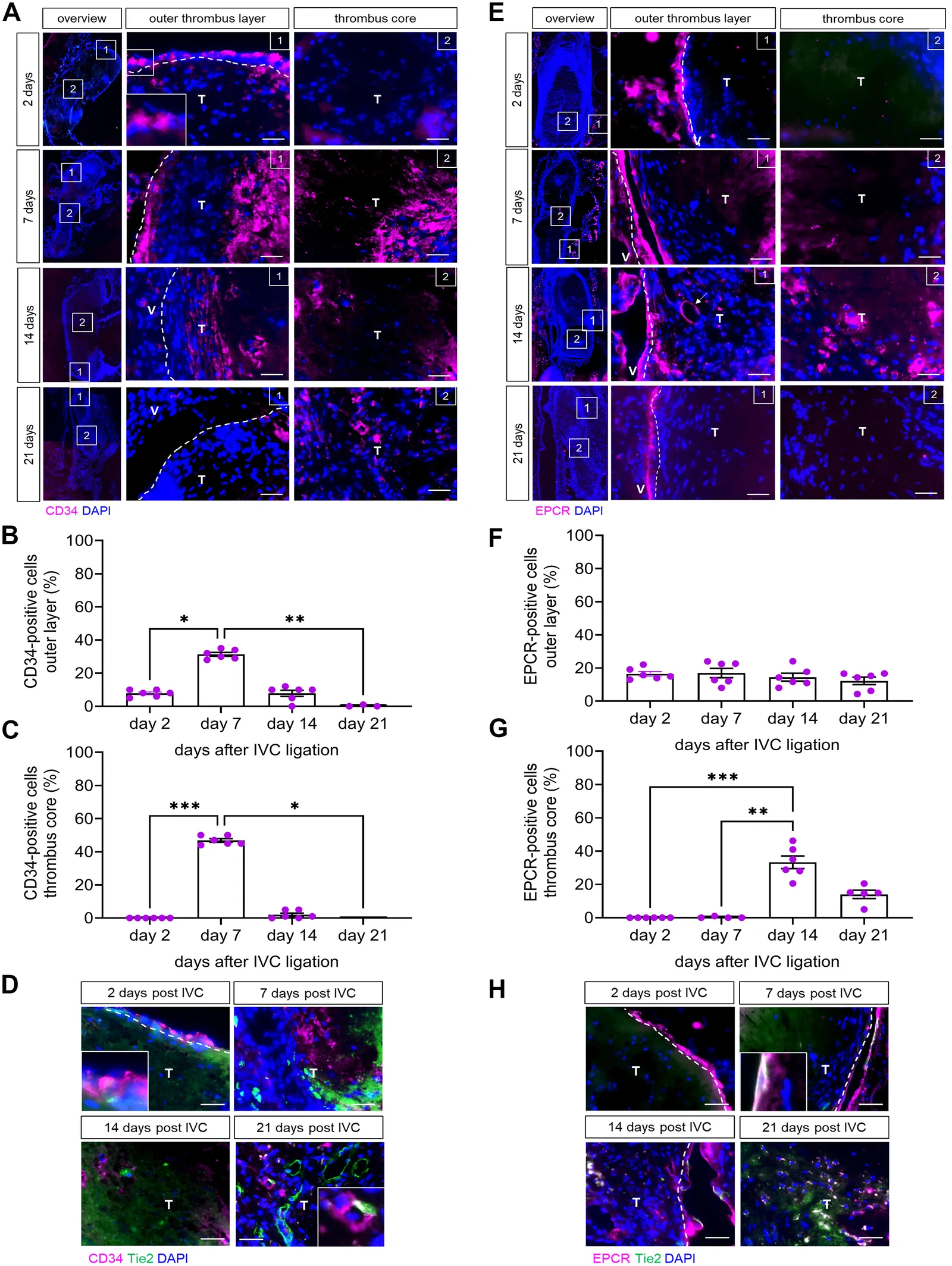
ECs re-express CD34 and endothelial protein C receptor (EPCR) during thrombus revascularization. Representative images (A) and quantitative analysis of venous thrombi isolated at different time points (as indicated) and stained for CD34 (purple signal) in the outer thrombus layer (B) and the thrombus core (C). Representative images (E) and quantitative analysis of venous thrombi isolated at different time points and stained for EPCR in the outer thrombus layer (F) and the thrombus core (G). Representative images of TIE2 reporter ECs stained for CD34 (D) or EPCR (H) at the indicated time points. V indicates the vein and T the thrombus. A scattered white line was added to outline the thrombus borders. Scale bars: 100 μm. ∗*P* < .05, ∗∗*P* < .01, ∗∗∗*P* < .001; 1-way anova, followed by Sidak multiple comparison test, in (F) and Kruskal–Wallis, followed by Dunn multiple comparison test, in (B), (C), and (G). Nonsignificant differences are not shown.

We next evaluated whether ECs residing in venous thrombus are of hematopoietic origin, using the type I transmembrane sialomucin CD34, expressed on mature ECs and hematopoietic stem cells, as a marker of vascular progenitor ECs involved in blood vessel formation [37]. CD34-immunopositive ECs started to appear at the outer layers of the thrombus early on day 2, and their numbers significantly increased on day 7, normalized on day 14, and were not detected on day 21 (Figure 3A, B). Similar findings were observed for the number of CD34-immunopositive cells within the thrombus core (Figure 3A, C). Dual staining of tissue sections from TIE2-endothelial reporter mice showed no or only minimal overlap of both signals and only an occasional colocalization in intrathrombotic vessels on day 21 (Figure 3D). These findings are in line with the expression of CD34 on hematopoietic stem and progenitor cells in the bone marrow and the conditional TIE2-mediated reporter gene activation at a later developmental stage.

Furthermore, we examined the expression of EPCR, a type I transmembrane glycoprotein primarily expressed on the surface of ECs of large blood vessels, which regulates coagulation via protein C activation, in cooperation with thrombomodulin [38,39]. Previous studies uncovered EPCR as a marker for vascular endothelial stem cells [40]. Interestingly, EPCR-immunopositive ECs were present at all time points after the IVC ligation, primarily in the outer layer of venous thrombus (Figure 3E, F), whereas EPCR expression levels in the thrombus core significantly increased later and on day 14 (Figure 3E, G). TIE2 lineage–derived endothelial cells coexpressing EPCR were seen lining the inner IVC on days 7 and 14 and vessel-like structures on day 21 (Figure 3H). This observation suggests that ECs reexpress progenitor markers during thrombus revascularization.

Regarding the signals recruiting ECs and their progenitors into the organizing venous thrombus, immunolabeling of CA IX as a surrogate marker for hypoxia showed a significant increase on day 7, at both the outer layer and the thrombus core, followed by normalization at days 14 and 21 (Supplementary Figure 4A–C). SDF1α immunosignals also increased on day 7 but continued to be elevated at later time points (Supplementary Figure 4D–F), supporting a possible role of both signaling pathways in the recruitment of inflammatory and progenitor cell during venous thrombus organization.

### 3.4 Pericytes appear after endothelial cells during thrombus organization

**Figure 4:**
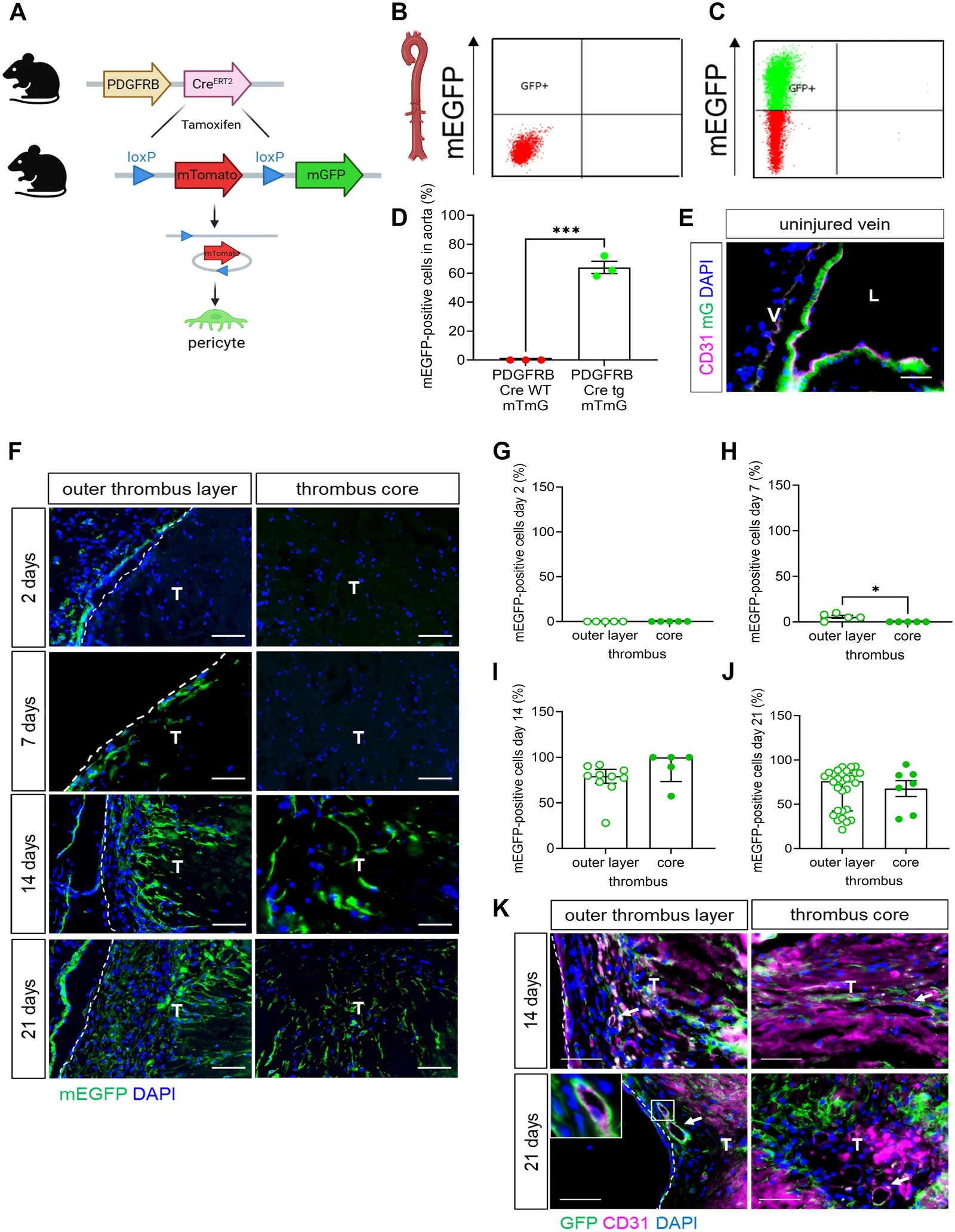
Pericytes appear after endothelial cells to support the newly formed vessel-like structures within organized venous thrombi. Schematic drawing showing the generation of tamoxifen-inducible pericyte (platelet-derived growth factor receptor beta [PDGFRB]) reporter mice (A). Flow cytometry dot blots of PDGFRB wild-type (WT) mTmG (B) and PDGFRB TG mTmG (C) mice and quantitative analysis (D) of the whole aorta. Representative fluorescence image of a cryopreserved uninjured vein from a PDGFRB reporter mouse stained using CD31 to visualize endothelial cells (purple signal; E). Representative images (F) and quantitative analysis (G–J) of PDGFR reporter–positive cells within venous thrombi isolated at different time points after the inferior vena cava ligation, as indicated. Representative images (K) from the outer thrombus layer (left panel) and thrombus core (right panel) isolated on day 14 and day 21 and stained to detect mEGFP–CD31 dual-positive cells. Scale bars: 100 μm. ∗*P* < .05, ∗∗∗*P* < .001; Student’s *t*-test in (D) and Mann–Whitney test in (G–J). Nonsignificant differences are not shown.

To answer the question whether ECs revascularizing venous thrombi are guided by pericytes or following pericyte sleeves [41], we examined the time course of their presence using mice expressing mTmG under control of the tamoxifen-inducible PDGFRB promoter [42] (Figure 4A). Flow cytometry of whole mouse aorta homogenates confirmed significantly increased numbers of mEGFP-positive cells in transgenic mice compared with those in WT control mice (Figure 4B–D). Strong fluorescent signals indicating PDGFRB reporter cells were seen immediately underneath the CD31-immunopositive cell layer lining the uninjured IVC (Figure 4E), confirming their denomination as pericytes. Time course analysis after the IVC ligation showed that PDGFRB–pericyte reporter cells in the IVC wall decreased on day 7 (Figure 4F–H) and that PDGFRB–pericyte reporter cells started to appear on day 14 (Figure 4F, I, J), that is later than the TIE2 reporter ECs (data shown in Figure 1F–J). Vascular structures lined by CD31-immunopositive cells and surrounded by pericyte reporter cells were seen only on day 21 (Figure 4K). These data are in line with pericytes supporting the newly formed vessel-like structures within the venous thrombus.

### 3.5 Erythrocyte lineage cells during venous thrombus formation and resolution

A characteristic feature of venous thrombi, as opposed to arterial thrombi, is the high presence of erythrocytes (RBCs) [43]. Their hemodynamic properties promote the margination of other blood cells and direct contact with the vessel wall. In this study, we asked the question whether RBCs play a role for the intrathrombotic distribution of endothelial and pericyte (progenitor) cells during thrombus organization. For this, we used mice expressing the mEGFP under the constitutive control of the EpoR promoter (Supplementary Figure 5A), expressed in early erythroid progenitor cells [21]. Flow cytometry confirmed significantly increased numbers of mEGFP-positive cells in whole blood of transgenic mice compared with those in WT control mice (Supplementary Figure 5B–D). Immunofluorescence microscopy analysis showed the presence of mEGFP-labeled RBCs in the vein lumen (Supplementary Figure 5E). Time course analysis of venous thrombus organization revealed strong erythrocyte reporter accumulation at all time points after the IVC ligation, near the vein wall and immediately attached to the inner vein EC layer on day 2, whereas unlabeled nucleated cells dominated this area and translocated erythrocyte signals toward the core of the thrombus at later time points (Supplementary Figure 5F–J). Immunostaining using CD45 as a pan-hematopoietic marker identified the nucleated cells at the outer thrombus layer and thrombus core as leukocytes (Supplementary Figure 5K), and CD45-positive cell numbers were significantly increased at day 2 (Supplementary Figure 5L, M). Intravital microscopy following IVC ligation in C57BL/6 WT mice, injected with either fluorescently labeled leukocytes or platelets or with erythrocytes from membrane Tomato transgenic mice (Supplementary Figure 6A), revealed that the recruitment of leukocytes was most pronounced and occurred within 5 minutes (Supplementary Figure 6B, E), followed by the appearance of platelets (Supplementary Figure 6D, E). On the contrary, a significant active recruitment of erythrocytes was not observed (Supplementary Figure 6D, E), in line with their distribution and passive accumulation at the outer thrombus layers according to their fluid dynamics and the law of Hagen-Poiseuille.

### 3.6 Histologic analysis of human pulmonary thrombus material at different organizational stages

**Figure 5:**
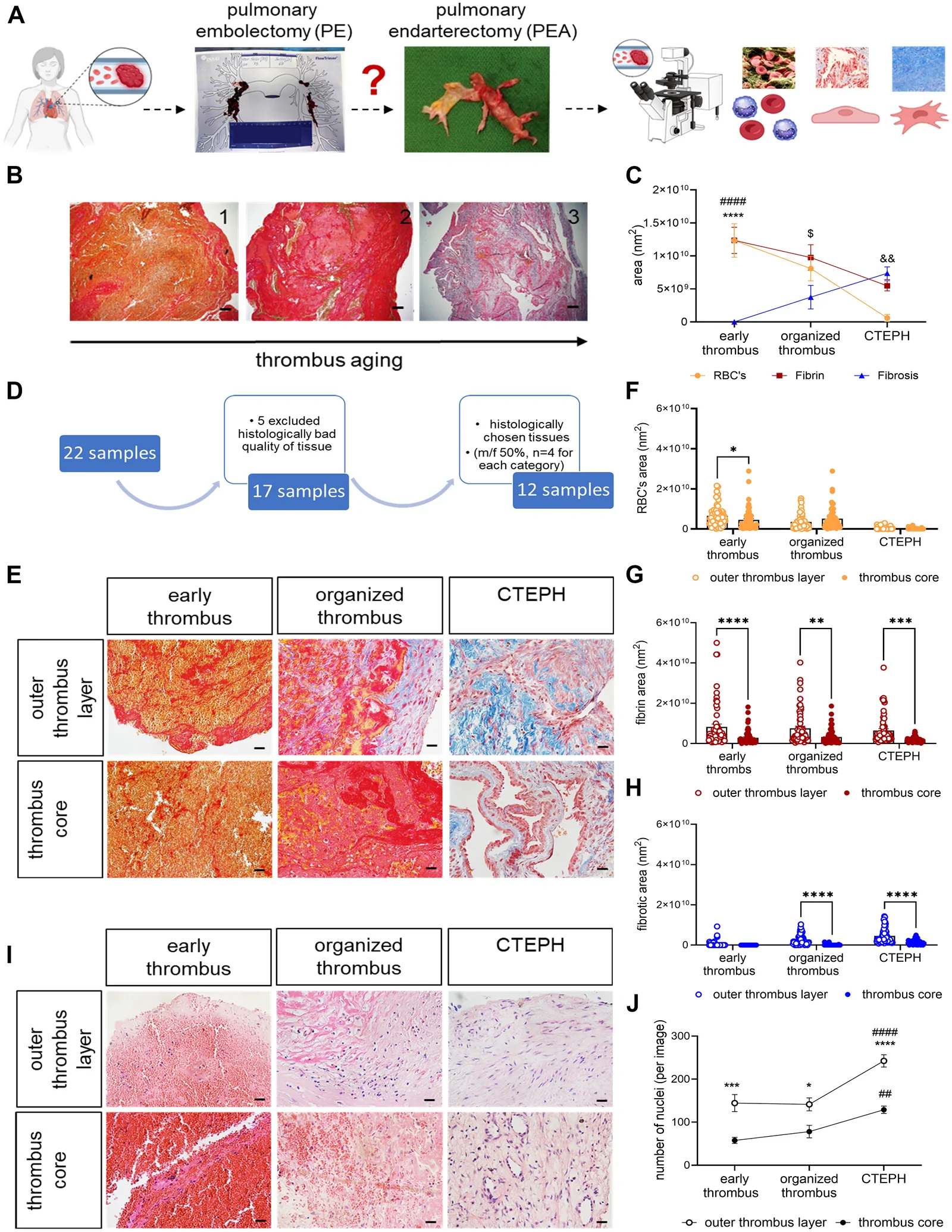
Organization of human pulmonary thromboemboli from fresh, red blood cell (RBC)-rich, and fibrin-rich thrombi in pulmonary embolism (PE) toward vascularized fibrotic thrombi in chronic thromboembolic pulmonary hypertension (CTEPH). Schematic drawing showing the design of the analysis of patients’ thrombotic tissue samples after pulmonary embolectomy and pulmonary endarterectomy (PEA) (A). Representative images showing 3 different stages of thrombus organization after Carstairs staining isolated via pulmonary embolectomy (1 and 2) and the chronic PEA material retrieved from patients with CTEPH (3) (B). Quantitative analysis of erythrocytes (RBCs), fibrin, and fibrosis in early and organized thrombi and in a CTEPH material (C). ∗∗∗∗*P* < .0001 and ####*P* < .0001 for RBCs and fibrin vs fibrosis; $*P* < .05 for fibrin vs fibrosis; and &&*P* < .01 for RBCs vs fibrosis; 2-way anova, followed by Sidak multiple comparison test. Graphical visualization of the selection of lung tissue samples for further analysis (D). Representative images (E) and quantitative analysis of RBCs (F), fibrin (G), and fibrosis (H) in the outer thrombus layer (upper panel) and the thrombus core (lower panel) of early, organized and chronic thrombi (CTEPH). ∗*P* < .05, ∗∗*P* < .01, ∗∗∗*P* < .001, ∗∗∗∗*P* < .0001; 2-way anova, followed by Sidak multiple comparison test. Representative images (I) and quantitative analysis (J) of changes in the number of nucleated cells during thrombus organization. ∗*P* < .05 and ∗∗∗∗*P* < .0001 vs thrombus core and ##*P* < .01 and ####*P* < .0001 vs values in the same group; 2-way anova, followed by Sidak multiple comparison test. Nonsignificant differences are not shown. Scale bars in (B), (E), and (I): 50 μm.

Finally, we asked whether the sequence of cellular events seen in experimental venous thrombus organization can be applied to characterize human venous thrombi of different ages and organizational stages (Figure 5A). For this, we analyzed pulmonary embolectomy samples from patients with acute PE and pulmonary endarterectomy samples from patients with CTEPH. Modified Carstairs’ staining revealed that PE thrombi from patients undergoing pulmonary embolectomy were rich in RBCs and fibrin and thus at an early organizational stage, as expected, whereas fibrotic tissue was detected only in 1 of 6 tissue samples (a 73-year-old man who presented with dyspnea for 6 days). On the contrary, RBCs were largely absent, while the amount of fibrotic tissue increased in PEA thrombi from patients with CTEPH (Figure 5B, C). Because these data showed a low prevalence of thrombi at an intermediate organizational stage, we expanded our analysis by including 12 postmortem tissue biopsies from patients who died at different time points after the diagnosis of PE and selected samples representing not only early and chronic but also in-between stages of thrombus organization (*n* = 4 per group) (Figure 5D). This analysis of these lung tissue specimens containing intact pulmonary vessels also allowed us to look at the outer thrombus layers and the thrombus core, similar to our murine model. Quantification of RBC numbers (Figure 5E, F), fibrin (Figure 5E, G), and fibrosis (Figure 5E, H) recapitulated the reduction of RBCs and fibrin and increase of fibrosis over time, most prominently at the outer thrombus layers. The number of nucleated cells also was significantly higher in the outer thrombus layer than in its core and further significantly increased from acute and organized vs CTEPH (Figure 5I, J). Of note, vessel-like structures were only seen in CTEPH samples. Our findings thus confirm thrombus organization from fresh, RBC-rich, and fibrin-rich thrombi in PE toward vascularized fibrotic thrombi in CTEPH.

### 3.7 Key markers identified in experimental murine deep vein thrombosis are expressed during human venous thrombus organization

**Figure 6:**
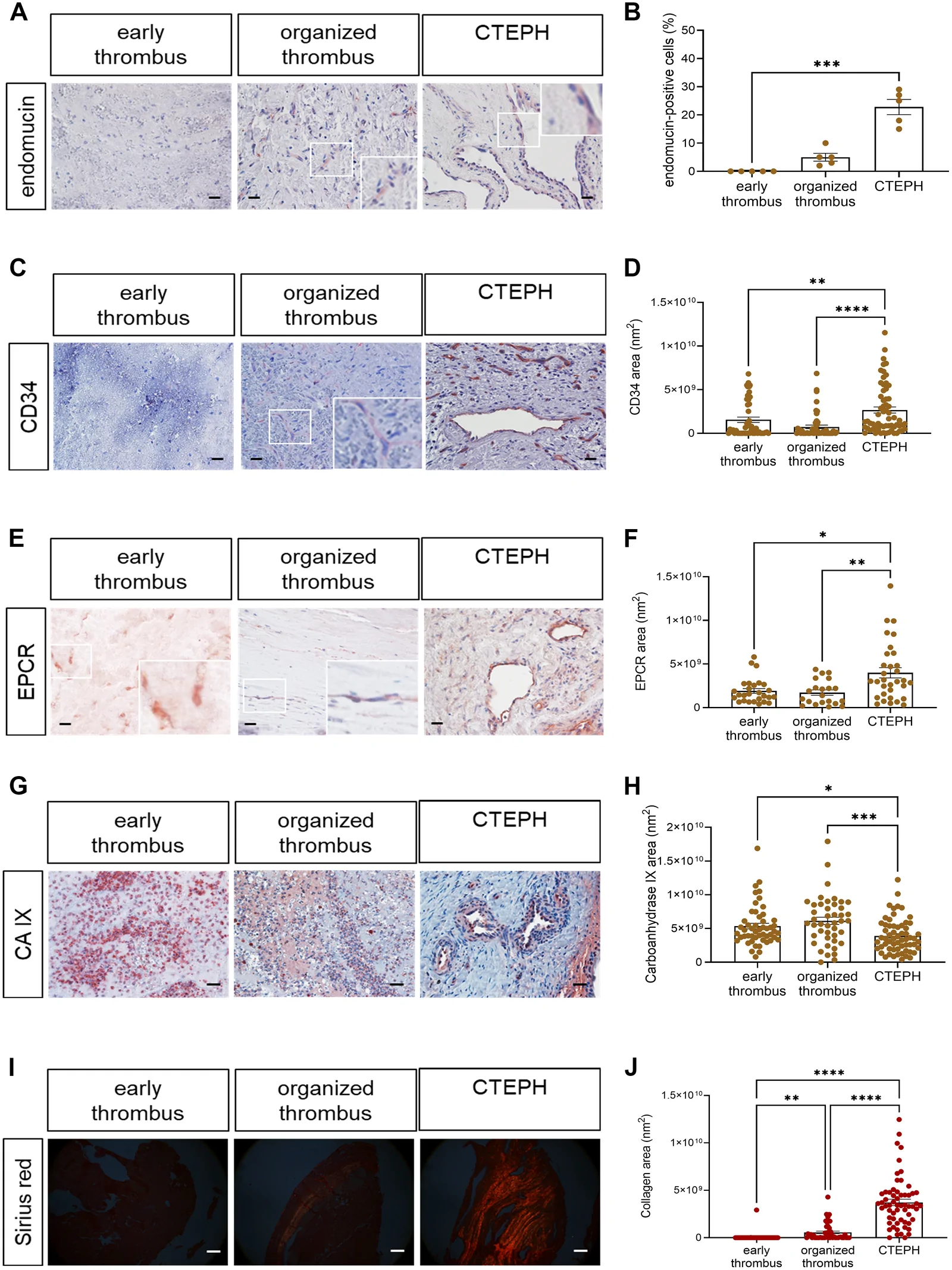
Key markers identified in experimental murine deep vein thrombosis are expressed during human thrombus organization. Representative images (A) and quantitative analysis (B) of endomucin-positive vascular structures within early and organized thrombi or chronic thrombi in chronic thromboembolic pulmonary hypertension (CTEPH). Representative images (C) and quantitative analysis (D) of CD34-positive vessels within early and organized thrombi or chronic thrombi in CTEPH. Representative images (E) and quantitative analysis (F) of endothelial protein C receptor (EPCR)-positive vessels within early and organized thrombi or chronic thrombi in CTEPH. Representative images (G) and quantitative analysis (H) of carboanhydrase (CA) IX expression in early and organized thrombi or chronic thrombi in CTEPH. Representative images (I) and quantitative analysis (J) of interstitial collagens (visualized using Sirius red under polarized light) during human venous thrombus organization. ∗*P* < .05, ∗∗*P* < 0.01, ∗∗∗*P* < .001, ∗∗∗∗*P* < .0001; Kruskal–Wallis, followed by Dunn multiple comparison test, in (B), (D), (H), and (J). Nonsignificant differences are not shown. Scale bars in (A), (C), (F), (G), and (I): 50 μm.

An important question is at which of these abovementioned organizational stages of human thrombi ECs and their progenitors can be observed. In this study, we made the interesting observation that endomucin-positive ECs were present already within organized human thrombi and that the numbers of endomucin-positive cells lining significantly increased in CTEPH specimens (Figure 6A, B). Similar findings were observed after labeling of CD34-positive cells (Figure 6C, D). Not only Endomucin- and CD34-positive but also EPCR-positive cells (Figure 6E, F) were found to line the irregular, enlarged intrathrombotic vessels characteristic of this disease. Immunolabeling using CA IX as a surrogate marker to detect hypoxic conditions within the thrombotic material revealed that CA IX–positive areas were largest at the early stages of thrombus formation and decreased in CTEPH (Figure 6G, H). This finding is in line with hypoxia as an important chemoattractant for early cell migration into the thrombus, as we have seen in mouse thrombi. Interestingly, CA IX–positive cells were observed surrounding vessel-like structures in CTEPH. The absence of interstitial collagen in early venous thrombi, as shown using Sirius red staining and polarized light, confirmed our classification and revealed that the highest collagen-positive area was present in CTEPH samples (Figure 6I, J).

## 4 Discussion

Our experimental findings in mice demonstrate that TIE2 reporter ECs can be identified within the organizing venous thrombus as early as day 7 after the IVC ligation and that the formation of vessel-like structures is complete on day 21. We show that TIE2 reporter ECs within the venous thrombus first express early endothelial markers (CD31) and progenitor cell markers (CD34 and EPCR), followed by the expression of VE-cadherin at a later stage. We also identified the expression of endomucin, a venous endothelial marker and functional coreceptor of VEGF receptor (VEGFR)2, as the most specific marker of venous thrombus angiogenesis. Hypoxia and overexpression of SDF1α may attract additional cells into the organizing thrombus, including progenitor and inflammatory cells. On the contrary, pericytes are recruited as supporting cells and appear within venous thrombi later than ECs. Although RBC cells were the most abundant cell type in venous thrombi, their intrathrombotic localization but not numbers changed over time. Comparison with human venous thrombi of different ages and organizational stages not only confirmed that RBCs are the most abundant cells in early and nucleated cells in chronic thrombi, such as CTEPH, but also revealed the presence of CA IX–positive cells and cells expressing endomucin, CD34, and EPCR already in early stages of thrombus organization.

The vascularization of venous thrombi is an important event during thrombus organization and contributes to the restoration of blood flow and vascular patency. Defective angiogenesis of venous thrombi is also a central mechanism of thrombus nonresolution, leading to chronic thrombofibrotic obstructions. This phenomenon is most prominently seen in the vasculature of the lung and leads to nonresolution of pulmonary emboli derived from deep veins and chronic pulmonary artery obstructions [14,15,44]. While previous studies have focused on angiogenic growth factors and downstream signaling events regulating venous thrombus angiogenesis, little is known about the time course of thrombus revascularization, subtype, and origin of ECs and whether they follow pericyte sleeves or invade the thrombus first.

Previous analyses of venous thrombus resolution in mice, including work from our group, used immunohistochemical techniques to detect endothelial and other cell types [18,[45], [46], [47]]. However, the expression of endothelial markers may change in response to alterations in blood flow, oxygen levels, or other damage signals. To avoid this important limitation, we used a genetic fate-mapping approach to irreversibly label TIE2 lineage–derived ECs and PDGFRB lineage–derived pericytes with mEGFP. Our data document the initial loss of TIE2 reporter ECs lining the IVC lumen, which is followed by their reappearance on day 7. In line with our observations, a recent single-cell sequencing analysis of murine venous thrombi, including the IVC wall, showed fewer ECs 24 hours after the deep vein thrombosis (DVT) [46]. This was paralleled by the differential regulation of genes related to angiogenesis and hypoxia [46]. Our analysis also revealed that TIE2 reporter ECs invade thrombi prior to measurable reductions in thrombus size or fibrin content. Time course analysis of experimental venous thrombus resolution using nanogold contrast-enhanced microcomputed tomography also showed a reduction in thrombus volume not earlier than day 14 [48]. At this time, intrathrombotic contrast agent extravasation was observed and interpreted as a consequence of immature neovascular channels. Vessel-like structures lined by cells expressing the TIE2-endothelial reporter and tight junction protein VE-cadherin also appeared late in our study, corroborating these earlier observations of leaky neovessels.

Venous thrombus formation in the IVC ligation model is driven by endothelial activation, whereas overt endothelial injury or loss was not observed [49]. Although electron microscopy showed intact ECs 6 hours after the IVC ligation [50], for later time points, this assumption is based on the detection of ECs using immunological methods. While we also observed a continuous lining of the inner IVC with CD31, TIE2 reporter ECs were lost on day 2 after the injury. We noted that most of the CD31-positive area did not contain nucleated cells, suggesting that CD31-positive platelets adhering to the denuded inner IVC layer may have produced this signal. Of note, the time point reported for the earliest appearance of neovessels within venous thrombi varies, ranging from as early as day 3 [9] to day 5 [51] and later [52,53], may reflect differences in the IVC ligation model, their definition, and the markers used to detect them.

Although we did not specifically label or detect platelets during thrombus organization in our study, they undoubtedly are major cellular players during venous thrombus formation, together with monocytes and neutrophils [50]. Interestingly, a previous detailed analysis of 48-hour-old venous thrombi in the IVC stenosis model showed that the white (platelet-enriched) part localizes to the distal end of the thrombi, whereas the red (RBC-enriched) part accumulates near the ligation site [54], suggesting that the reduction of thrombus length seen in our model beyond day 14 may reflect platelets at work. Another angiogenic factor released from activated platelets is SDF1α, and we found that the expression of this hematopoietic cytokine within venous thrombi increased during the early organizational stages and at the same time when progenitor cell numbers significantly increased. Although these data would support its role in the recruitment, migration, and retention of cells within the thrombus, as shown earlier in mouse models of ischemia-induced neovascularization [55,56], bone marrow transplantation experiments would have been necessary to definitely answer this question.

Endothelial activation promotes the recruitment of platelets and immune cells to the site of injury via the upregulation of adhesion molecules, including CD31 and ICAM2 [57,58]. While CD31 expressing ECs were clearly present, ICAM2 expression was unexpectedly not observed in the thrombosed IVC segment but rather in the vessels of the adventitia and perivascular space, as well as the inner lining of the adjacent aorta. These observations support the concept of a general endothelial activation and the clinical, as well as experimental, evidence connecting DVT with arteriosclerosis [[59], [60], [61], [62]].

Our analyses identified the endothelial glycocalyx glycoprotein endomucin as early and specific marker for ECs revascularizing venous thrombi. Endomucin is highly expressed on venous endothelium and therefore used as its marker [34], although other EC types also express endomucin [63]. Our findings are in line with a recent genetic fate-mapping study identifying venous ECs as the primary endothelial subtype driving the expansion of the angiogenic network during pathological angiogenesis in a mouse model of oxygen-induced retinopathy [64]. The central role of VEGF and VEGFR signaling for venous thrombus revascularization has been shown in previous studies [11,12]. Interestingly, VEGFR2-positive cells also represent a common precursor for ECs and hematopoietic progenitor cells [65], which, in response to VEGF, acquire characteristics of endothelial lineage cells and give rise to VE-cadherin–positive ECs during further differentiation steps [66]. This sequence of differentiation events aligns well with our observation of changes in EC marker expression over time.

The recruitment of cells expressing CD34 or EPCR, markers of hematopoietic stem and endothelial progenitor cells, from the bone marrow into resolving venous thrombi is known for some time and was shown to occur between day 7 and day 14 after IVC ligation [67]. Notably, and in contrast to murine venous thrombi, we observed the presence of EPCR-positive cells already in thrombus material obtained from patients with acute PE, and they expanded and were seen lining intrathrombotic vessels during thrombus aging. Sirius red staining of interstitial collagens excluded the possibility that thrombi were at a more advanced organizational stage. Interestingly, no GFP expressing cells lining vascular channels were found at these time points using constitutive TIE2 reporter bone marrow mice [67]. In our study, using mice with inducible TIE2 mEGFP reporter gene expression, cells lining intrathrombus vessels expressing the reporter gene also were not seen before day 21. The importance of TIE2-expressing EPCs for revascularization was shown during physiological (ovulation) and pathological (wounding, tumor growth, hindlimb ischemia, and myocardial infarction) angiogenesis [68], whereas our data are the first to report their presence during venous thrombus organization. Regarding the possibility of Cre-mediated reporter gene activation in TIE2-positive hematopoietic cells, the Cre recombinase expressed in the transgenic mice examined in our study contains an endothelial-specific enhancer which allows it to target reporter gene expression specifically and uniformly to virtually all vascular ECs throughout embryogenesis and adulthood [69]. Temporal Cre activity induction in adult mice further minimized the possibility of Cre-mediated gene recombination in hematopoietic cells. Nevertheless, it should be noted that TIE2 lineage tracing and the use of markers cannot distinguish with certainty between resident ECs, circulating progenitor cells, or hematopoietic TIE2-positive populations and that our study therefore does not establish the precise cellular origin of intrathrombotic ECs. In future studies, the use of EC subtype–specific Cre mouse lines irreversibly labeling tip and sprout cells [64] would be of interest to dissect the angiogenic process during thrombus organization.

In summary, using a murine venous thrombosis model and pulmonary embolectomy and endarterectomy samples or postmortem lung tissue from patients with thrombi at different organizational stages occluding the pulmonary vasculature, we showed that TIE2 reporter ECs are detectable within murine thrombi as early as day 7 and that their numbers increase over time to form vessel-like structures later. ECs within thrombi transiently express the stem and progenitor markers such as CD34 and EPCR, and hypoxia and hematopoietic cytokines might aid the recruitment of not only progenitor but also immune and inflammatory cells to the thrombotic environment. PDFGRB lineage cells appear later and follow ECs. We and others have previously shown that thrombotic material isolated from patients with CTEPH consists of diverse histologically organized compartments [14,18]. In this study, we used a genetic fate-mapping approach to irreversibly label ECs, pericytes, and RBCs to follow their presence and expansion during venous thrombus revascularization. We acknowledge the fact that the extent of not only fibrosis but also other events during thrombus organization are determined by the type of injury model [70] and that those findings may be different in alternative models of venous thrombosis [[71], [72], [73], [74]]. Moreover, the human data derived from a heterogeneous group of acute PE, postmortem thrombi, and chronic CTEPH material provide supportive histologic correlation rather than direct temporal validation of findings in the murine models. Nevertheless, a better understanding of venous thrombus organization might provide a mechanistic basis for catheter-based revascularization strategies and help to improve clinical procedural outcomes. Targeting thrombus angiogenesis may also reduce the requirement for thrombolysis with expected reductions in hemorrhagic complications.

A limitation of the current study is the potential selection bias of endothelial-specific markers to identify the diverse cell populations. In the future, single-cell RNA sequencing could help to more comprehensively characterize the transcriptional profiles of the cells residing within venous thrombi. Moreover, and as a caveat, thrombus material from human acute pulmonary emboli and CTEPH is distinct from resolving murine DVT tissue.
