# Supplementary material for: Visualization of new vessel formation during venous thrombus organization using genetic fate mapping
Source: Res Pract Thromb Haemost. 2026 Jul 14;10(5):106842. doi: 10.1016/j.rpth.2026.106842 (PMC13507550; doi:10.1016/j.rpth.2026.106842)
Supplement: Supplementary file 1 [file mmc2.docx]

**Table S1. Primer sequences for mouse genotyping**

| **Gene** | | **Primer sequences**  (in 5‘– 3‘direction) | **T_m_**  (°C) | **cycles** | **ref** |
| --- | --- | --- | --- | --- | --- |
| ***ErGFP.Cre*** | F: GTGTGGCTGCCCCTTCTGCCA  F: GGCAGCCTGGGCACCTTCAC  R: CAGGAATTCAAGCTCAACCTC | | 60 | 40 | E[1] |
| ***PDGFRB.CreER^T2^*** | F: GAACTGTCACCGGGAGGA  F: CAAATGTTGCTTGTCTGGTG  R: GTCAGTCGAGTGCACAGTTT  F: AGGCAAATTTTGGTGTACGG | | 60 | 28 | E[2] |
| ***ROSA26mTmG*** | F: TAGAGCTTGCGGAACCCTTC  F: AGGGAGCTGCAGTGGAGTAG  R: CTTTAAGCCTGCCCAGAAGA | | 60 | 28 | E[3] |
| ***Tie2.CreER^T2^*** | F: CGAGTGATGAGGTTCGCAAG  R: TGAGTGAACGAACCTGGTCG | | 58 | 35 | E[4] |

**Table S2. Time course analysis of venous thrombus revascularization in lineage tracing mice**

|  | uninjured | day 2 | day 7 | day 14 | day 21 |
| --- | --- | --- | --- | --- | --- |
| EpoR mEGFP | 0 | 0 | 0 | 0 | 0 |
| TIE2 mEGFP | + | 0 | ++ | ++ | ++ |
| PDGFRB mEGFP | 0 | 0 | 0 | +++ | ++ |

0, not detected; +, weak; ++, moderate; +++, strong

**Table S3. Semi-quantitative analysis of changes in endothelial antigen expression on cells lining the inner layer of the inferior Vena cava in response to ligation**

| EC marker | uninjured | day 2 | day 7 | day 14 | day 21 |
| --- | --- | --- | --- | --- | --- |
| CD31 | ++ | + | 0 | + | ++ |
| VE-cadherin | ++ | ++ | 0 | +++ | ++ |
| EMCN | ++ | + | ++ | ++ | ++ |
| IB4 | ++ | + | 0 | 0 | + |
| ICAM2 | 0 | 0 | 0 | 0 | 0 |

0, not detected; +, weak; ++, moderate; +++, strong

**Table S4. Semi-quantitative analysis of endothelial marker expression in cells covering the outside of the IVC thrombus**

| EC marker | uninjured | day 2 | day 7 | day 14 | day 21 |
| --- | --- | --- | --- | --- | --- |
| CD31 | n.a. | 0 | 0 | 0 | +++ |
| VE-cadherin | n.a. | + | ++ | +++ | +++ |
| EMCN | n.a. | + | +++ | +++ | +++ |
| IB4 | n.a. | + | 0 | 0 | + |
| ICAM2 | n.a. | 0 | 0 | 0 | 0 |

0, not detected; +, weak; ++, moderate; +++, strong; n.a. not applicable.

**References**

1.	Heinrich, A.C., R. Pelanda, and U. Klingmuller, *A mouse model for visualization and conditional mutations in the erythroid lineage.* Blood, 2004. **104**(3): p. 659-66.

2.	Eilken, H.M., et al., *Pericytes regulate VEGF-induced endothelial sprouting through VEGFR1.* Nat Commun, 2017. **8**(1): p. 1574.

3.	Muzumdar, M.D., et al., *A global double-fluorescent Cre reporter mouse.* Genesis, 2007. **45**(9): p. 593-605.

4.	Forde, A., et al., *Temporal Cre-mediated recombination exclusively in endothelial cells using Tie2 regulatory elements.* genesis, 2002. **33**(4): p. 191-197.
